# Re-visiting the detection of porcine cysticercosis based on full carcass dissections of naturally *Taenia solium* infected pigs

**DOI:** 10.1186/s13071-017-2520-y

**Published:** 2017-11-16

**Authors:** Mwelwa Chembensofu, K. E. Mwape, I. Van Damme, E. Hobbs, I. K. Phiri, M. Masuku, G. Zulu, A. Colston, A. L. Willingham, B. Devleesschauwer, A. Van Hul, A. Chota, N. Speybroeck, D. Berkvens, P. Dorny, S. Gabriël

**Affiliations:** 1 Department of Paraclinical Studies, School of Veterinary Medicine, University of Zambia, P.O. Box 32379, Lusaka, Zambia; 2 Department of Clinical Studies, School of Veterinary Medicine, University of Zambia, P.O. Box 32379, Lusaka, Zambia; 3 Faculty of Veterinary Medicine, Ghent University, Salisburylaan 133, 9820 Merelbeke, Belgium; 4 One Health Center for Zoonoses and Tropical Veterinary Medicine, Ross University School of Veterinary Medicine, P.O. Box 334, Basseterre, St Kitts Saint Kitts and Nevis; 5 Department of Biomedical Sciences, Institute of Tropical Medicine, Kronenburgstraat 25, 2000 Antwerp, Belgium; 6 District Medical Office, Ministry of Health, P.O. Box 30205, Lusaka, Zambia; 7 Global Alliance for Livestock Veterinary Medicines, P.O. Box 52773 – 00100, Valley Arcade, Nairobi, Kenya; 8 Department of Public Health and Surveillance, Scientific Institute of Public Health (WIV-ISP), Rue Juliette Wytsman 14, 1050 Brussels, Belgium; 9 Université catholique de Louvain, Institute of Health and Society (IRSS), School of Public Health, 1200 Woluwe-Saint-Lambert, Brussels, Belgium

**Keywords:** *Taenia solium*, Cysticercosis, Pig carcass, Dissection, Liver, Antigen ELISA, Tongue palpation

## Abstract

**Background:**

*Taenia solium* is a neglected zoonotic parasite. The performances of existing tools for the diagnosis of porcine cysticercosis need further assessment, and their shortcomings call for alternatives. The objective of this study was to evaluate the performance of tongue palpation and circulating antigen detection for the detection of porcine cysticercosis in naturally infected pigs of slaughter age compared to full carcass dissections (considered the gold standard). Additionally, alternative postmortem dissection procedures were investigated. A total of 68 rural pigs of slaughter age randomly selected in the Eastern Province of Zambia were dissected. Dissections were conducted on full carcasses (or half carcass in case cysticerci were already detected in the first half), including all the organs. Total cysticercus counts, location and stages were recorded and collected cysticerci were identified morphologically and molecularly. All sera were analysed with the B158/B60 antigen detecting ELISA (Ag-ELISA).

**Results:**

Key findings were the high occurrence of *T. solium* infected pigs (56%) and the presence of *T. solium* cysticerci in the livers of 26% of infected animals. More than half of the infected carcasses contained viable cysticerci. Seven carcasses had *T. hydatigena* cysticerci (10%), out of which five carcasses were co-infected with *T. hydatigena* and *T. solium*; two carcasses (3%) had only *T. hydatigena* cysticerci. Compared to full carcass dissection, the specificity of the Ag-ELISA to detect infected carcasses was estimated at 67%, the sensitivity at 68%, increasing to 90% and 100% for the detection of carcasses with one or more viable cysticerci, and more than 10 viable cysts, respectively. Tongue palpation only detected 10% of the cases, half carcass dissection 84%.

Selective dissection of the diaphragm, tongue and heart or masseters can be considered, with an estimated sensitivity of 71%, increasing to 86% in carcasses with more than 10 cysticerci.

**Conclusions:**

Depending on the aim of the diagnosis, a combination of Ag-ELISA and selective dissection, including investigating the presence of *T. hydatigena*, can be considered. Full carcass dissection should include the dissection of the liver, kidneys, spleen and lungs, and results should be interpreted carefully, as small cysticerci can easily be overlooked.

## Background

*Taenia solium*, also known as the pork tapeworm, is a zoonotic parasite which is endemic in developing countries of Africa, Asia and Latin America where pigs are raised as a food source and are kept under free range conditions [1]. The tapeworm causes two disease conditions in humans, taeniosis (intestinal tapeworm infection) and cysticercosis (metacestode larval stage infection). Infection of the central nervous system with cysticerci leads to a condition known as neurocysticercosis (NCC), which is the most prevalent helminthic infection of the nervous system and a leading cause of acquired epilepsy worldwide [2]. Porcine cysticercosis is acquired when pigs get access to human faeces containing *T. solium* ova. Cysticerci are primarily located in muscle tissues and the brain [1]. This condition may lead to carcass condemnations and hence extensive economic losses which can be significant (50–60% of the carcass value) in some countries [3, 4].

Zambia is endemic for *T. solium* infection with prevalences of, porcine cysticercosis ranging from 14% (Ag-ELISA) to 64% (Bayesian estimation) [5, 6]; human cysticercosis between 5.8–15% (Ag-ELISA); and human taeniosis between 6.3–12% (Copro-Ag ELISA) [7, 8]. Recent results indicated over 50% of cases of acquired epilepsy (late onset) are due to NCC [9]. There is therefore an urgent need to control and possibly eliminate the parasite in Zambia.

A number of control strategies, such as human and/or pig treatment, pig vaccination, health education (reviewed by [10]) have been evaluated by observing the changes in prevalence of porcine cysticercosis through diagnostic procedures like tongue palpations, routine meat inspection, serological techniques and carcass dissections. Performances of these tests have been estimated (using a Bayesian approach, confirmed using dissection results of 65 pigs) at 21% sensitivity (Se) and 100% specificity (Sp) for tongue palpation and 22% Se and 100% Sp for meat inspection [6, 11]. Serological techniques, detecting circulating antigens and specific antibodies are frequently used, although test performances are a matter of debate and need further assessment. The monoclonal antibody-based circulating antigen detecting ELISAs have been reported to have a Se of 65–93% and a Sp of 70–100% in detecting porcine cysticercosis in naturally infected pigs [6, 12]; for the detection of specific antibodies the best performances are obtained with the enzyme-linked immunoelectrotransfer blot (EITB), with a sensitivity of 78–100% and a specificity of 43–100% [13, 14]. Carcass dissection (in 5 mm slices) is generally considered the gold standard diagnostic method in assessing infection status. It can describe the infection intensity, and stage, size and distribution of cysticerci in different organs [6, 11]. The major disadvantages of full carcass dissection are, the costs involved in purchasing of pigs, the labor, the time needed, and the need for trained personnel to conduct the dissections. To reduce labor and time, many researchers aim for half carcass dissections, though the performance of this method as a diagnostic tool needs to be assessed thoroughly. An additional disadvantage of carcass dissection is that the animals are removed from the study population, which can impact on the results in follow up studies of control interventions. As such, relatively few dissections have been done on naturally infected pigs in sub-Saharan Africa [6, 15, 16].

Clearly there is a need to assess the performances of currently available diagnostic tools for porcine cysticercosis, using full carcass dissection as a reference method, and to define valid alternatives to full carcass dissections.

The objective of this study was to evaluate the performance of tongue palpation and circulating antigen detection for the detection of porcine cysticercosis in naturally infected pigs of slaughter age via comparison with full carcass dissections. Additionally, alternative postmortem dissection procedures, such as half carcass dissection and specific organ/muscle group dissections, were investigated for their effectiveness to detect infected animals.

## Methods

### Study site

The CYSTISTOP Project is currently implementing measures for elimination/control of *Taenia solium* cysticercosis in Nyembe and Mtandaza rural communities of Katete and Sinda Districts, in the Eastern Province of Zambia. A baseline study was conducted between October and November 2015 in the study areas to establish the baseline prevalence of porcine cysticercosis through full carcass dissection (half carcass dissection if cysticerci were already identified in the first half of the carcass). The two districts were selected following the high prevalence of porcine and human cysticercosis/taeniosis reported by previous studies [5, 8, 9, 11]. Other factors considered were the accessibility of the areas and willingness to participate of the village representatives (village headmen) and health/veterinary officers.

### Sample size and pig selection

The sample size of pigs selected for postmortem examinations was calculated for the intervention study (CYSTISTOP, see above), and was based on 80% power to detect an effect of intervention using a one-sided likelihood ratio test at the 5% significance level with an assumption for prevalence of 20% before interventions. Calculations of the sample size were done using SAS 9.3, with the TWOSAMPLEFREQ command in the PROC POWER procedure (sample size = 67).

A list of pig households (HH) in the study area was drawn in excel, from which a random list of HH was generated (random function in excel) and pigs were purchased (one pig per HH). All pigs of slaughter weight and age from the selected HH, and to which the owner agreed to sell, were given arbitrary numbers. Random sampling was done by casting a dice and the number at which the dice settled was noted, the pig with that corresponding number was selected and purchased.

### Tongue palpation and euthanasia

Tongue examination/palpation was performed before slaughter by placing each pig in left lateral recumbence, where it was firmly restrained by three people. The mouth was opened by a wooden rod and the tongue pulled gently with a mutton cloth [6, 11]. Pigs were considered positive for cysticercosis if cyst-like nodules were either seen or felt [14] on any part of the tongue. Animals were slaughtered by a captive bolt gun, and thereafter exsanguinated.

### Blood collection, sera separation and storage

Immediately after exsanguination, blood was collected from either the jugular or the cranial vena cava veins in 50 ml falcon tubes. To maximize the amount of serum collected, the blood was placed in the refrigerator at 4 °C overnight. The following day, the blood was centrifuged at 3000× *rpm* for 15 min. Sera were separated and aliquoted in duplicate cryovials and stored at -20 °C until use.

### Carcass dissection and cysticercus counting

Carcasses and heads were skinned and the muscles separated from the bones. Muscle tissues/organs were excised from one carcass half of each pig, together with the complete head (eyes and brain) heart, tongue, neck, diaphragm, psoas muscles, spleen, kidneys, lungs and liver. Slices of maximum 0.5 cm thickness were made and cysticerci were enumerated. If cysticerci were identified in the half carcass, the cysticercus counts for the muscles of the half carcass were doubled and added to the counts of the complete head, heart, tongue, neck, diaphragm, psoas muscles, spleen, kidneys, lungs, liver, eyes and brain to obtain total carcass counts. If no cysticerci were found in the first half of the carcass, then the other half of the carcass was dissected (= full carcass dissection). When the number of cysticerci was too high to accurately count, 100 cysticerci per muscle/organ part was assumed.

Cysticerci were classified as viable if they had a translucent fluid with a visible whitish protoscolex; degenerated if they had a collapsed and viscous damaged cysticercus wall and absence of the cystic fluid; or calcified if they were non-cystic and with yellowish caseous masses [11, 16]. These were observed macroscopically and recorded on pig dissection spread sheets. *Taenia hydatigena* cysts were also looked for in the peritoneal cavity, on intestinal surfaces and in the visceral organs (livers, lungs, heart, kidney, diaphragm and omentum).

### Cysticercus viability test

A few cysticerci (1–4, depending on availability) were collected from all positive carcasses, washed in phosphate buffered saline (PBS) and put onto Petri dishes containing a 1:1 mixture of pig bile and normal saline, as described by [14]. They were incubated at room temperature for 2 h to allow for complete evagination of cysticercus scolices.

### Laboratory analysis of serum samples

The B158/B60 sandwich enzyme-linked immunosorbent assay (Ag-ELISA) was performed on sera to detect circulating antigens as described by Dorny et al. [6]. The status of the test samples was determined by comparing their optical densities to those of 8 negative control sera (from Zambian pigs) at a probability (*P*) < 0.001 [17].

### PCR-restriction fragment length polymorphism (PCR-RFLP) analysis

A number of cysticerci (at least 1) detected in the muscles were collected from all dissection-positive carcasses for confirmation by PCR-restriction fragment length polymorphism (PCR-RFLP) as described by Dermauw et al. [18]. Cysticerci detected in organs (e.g. liver, spleen and lungs) were also collected separately for each organ for molecular confirmation by PCR-RFLP.

### Data management and analysis

Data were entered into EpiData twice and statistical analyses were performed in STATA/IC 14.1 (StataCorp LP, College Station, TX, USA). Pigs were considered *T. solium* positive if at least one cysticercus was detected and confirmed as *T. solium*. Univariate logistic regressions were performed to evaluate differences between age (6–12 months *vs* ≥ 13 months), region, and sex. The sensitivity of using one (multiple) organ(s) to detect *T. solium* (an organ was considered positive when at least one *T. solium* cysticercus was detected) was estimated relative to whole carcass dissection results as criterion standard. Similarly, the sensitivity and specificity of the Ag-ELISA were estimated relative to whole carcass dissection results, considering a carcass as positive when (i) at least one *T. solium* cysticercus was detected; (ii) at least ten *T. solium* cysticerci were detected; (iii) at least one viable *T. solium* cysticercus was detected; (iv) at least ten viable *T. solium* cysticerci were detected. When determining the sensitivity and specificity of tests, exact binomial 95% confidence intervals were calculated according to Clopper & Pearson [19]. To evaluate the relation between the number of cysticerci and the Ag-ELISA ratio, the animals were grouped according to the total number of cysticerci: group 0 (between 1 and 10 cysticerci); group 1 (between 11 and 99 cysticerci) and group 2 (≥ 100 cysticerci). The Ag-ELISA ratios between each of the groups were compared using Wilcoxon rank-sum tests and the trend across the ordered groups was evaluated according to Cuzick [20]. The same was done to evaluate the relation between the number of viable cysticerci and Ag-ELISA ratio.

## Results

### Overall dissection results

A total of 68 pigs were selected and purchased for dissections (Nyembe, *n* = 37; Mtandaza, *n* = 31). All pigs dissected were local breeds indigenous to the Eastern Province of Zambia.

Cysticerci were detected by dissection in a total of 38 carcasses (56%), of which 32 were detected during the dissection of the first half of the carcass. An additional six cases were detected only during the dissection of the second half of the remaining 36 carcasses (full carcass dissection). As such, half carcass dissection would have picked up 84% (95% CI: 69–94%) of the infected carcasses. *Taenia solium* cysticerci were found in 4/68 (6%) pigs by tongue palpation. Tongue palpation detected 4 out of the 38 (11%; 95% CI: 2.9–25%) dissection positives.

No significant difference was determined in infection status between male (66% infected, 21/32) and female pigs (49% infected, 17/35, the sex of one animal was not noted) (OR = 2.0; 95% CI: 0.7–5.4, *P* = 0.162). In Nyembe and Mtandaza, 46% and 68% of pigs were *T. solium* positive, respectively, though the difference between both areas was not significant (OR = 0.4; 95% CI: 0.1–1.1, *P* = 0.074).

In the two age group categories; 6–12 months of age and ≥ 13 months, the infection occurrences were determined at 59% (24/41) and 52% (14/27), respectively, no significant differences were observed (OR = 1.3; 95% CI: 0.5–3.5, *P* = 0.587).

*Taenia hydatigena* cysticerci were identified in seven carcasses, representing 10% (7/68) of the total carcasses dissected, and all of these cysticerci were viable. Of the seven *T. hydatigena* positives, five carcasses were co-infected with *T. hydatigena* and *T. solium*. The other two carcasses (3%) had only *T. hydatigena* cysticerci. A total of 10 *T. hydatigena* cysticerci were observed in those seven carcasses.

### Infection intensity

The number of cysticerci per carcass dissected ranged from 1 to > 10,000. A high proportion of infected carcasses (76%) had low to moderate infection levels (42% with ≤ 10 cysticerci and 34% with 11–50 cysticerci). High infection levels of > 100 cysticerci were observed in 21% of infected carcasses, of which one carcass had > 10,000 cysticerci (exact number of cysticerci was impossible to count) (Table 1). In one carcass, very small (2 mm) cysticerci were detected. The majority of these small cysticerci had intact walls, semitransparent membrane and transparent cysticercus fluids. Several of these small cysticerci from this pig were subjected to the viability test as well and all of them evaginated, thus demonstrating their viability. The viable cysticerci collected from the carcasses and subjected to viability tests also evaginated within two hours.

**Table 1:** *Taenia solium* infection levels in infected carcasses

No. of cysticerci in carcass	No. of carcasses (%)
≤ 10	16 (42)
11–50	13 (34)
51–99	1 (3)
≥ 100	7 (18)
> 10,000	1 (3)
Total	38 (100)

### Distribution and classification of *T. solium* cysticerci

In 24% of the infected carcasses only viable cysticerci were detected, in 11% only degenerated cysticerci and in 16% only calcified cysticerci. In 13 (34%) of the infected carcasses, cysticerci of various developmental stages were observed in the same carcass, and all three stages were present in seven carcasses.

The distribution and number of *T. solium* cysticerci in different locations are shown in Table 2. The carcass with more than 10,000 cysticerci has not been included in Table 2, as no exact counts were conducted. A total of 3382 cysticerci were detected in the different tissues and organs of the 37 *T. solium* infected carcasses. Of the 3382 cysticerci detected, 2925 (86.5%) were viable, 127 (3.8%) were degenerated and 330 (9.8%) were calcified. In 4 carcasses, cysticerci were localized only in one organ/tissue in each case (pig 29: liver; pig 31: heart; pig 33: hind leg; pig 39: tongue). The brain recorded the lowest number of cysticerci (107), however most of them were viable (102/107; 95.3%). The liver recorded the lowest percentage of viable cysticerci (110/151; 72.9%) and highest percentage of calcified cysticerci (34/151; 22.5%). The foreleg recorded the highest number of *T. solium* cysticerci (1003).

**Table 2:** Distribution and stage of the total number of *Taenia solium* cysticerci detected in the different muscles/organs in 37 infected carcasses

Muscle/organ	Cysticercus stage			Total cysticerci/organ (%)
	Viable	Degenerated	Calcified	
Masseter	145	2	11	158 (4.7)
Heart	132	2	11	145 (4.3)
Tongue	153	1	18	172 (5.1)
Psoas	177	3	17	197 (5.8)
Diaphragm	187	5	23	215 (6.4)
Brain	102	0	5	107 (3.2)
Foreleg	883	22	98	1003 (29.7)
Hind leg	657	40	94	791 (23.4)
Liver	110	7	34	151 (4.5)
Other	379	45	19	443 (13.2)
Total (%)	2925 (86)	127 (4)	330 (10)	3382 (100)

*Note:* The carcass with more than 10,000 viable cysticerci is not included in the table

In this study, ten out of 38 infected carcasses had cysticerci in the liver (26%; 95% CI: 13–43%), one carcass had a cysticercus in the spleen (2.6%; 95% CI: 0.6–14%) and two carcasses had cysticerci in the lungs (5.3%; 95% CI: 0.6–18%).

In almost all the positive cases, *T. solium* cysticerci were detected in at least one or more predilection sites, except the one carcass that only had cysticerci in the liver. In carcasses infected with 1–10 cysticerci, most carcasses had cysticerci in the foreleg. For infected carcasses with 11–99 cysts, most carcasses had cysticerci in the hind and foreleg (Table 3).

**Table 3:** Number of carcasses with *Taenia solium* cysticerci in the specified muscle/organ of infected carcasses according to the overall infection level of the pig

Muscle/organ	Infection level (total number of *T. solium* cysticerci/carcass)			Total (*n* = 38)^a^
	1–10 cysticerci (n = 16)^a^	11–99 cysticerci (n = 14)^a^	≥ 100 cysticerci (*n* = 8)^a^	
Masseter	1	5	7	13
Heart	2	4	8	14
Tongue	4	6	7	17
Psoas	0	2	8	10
Diaphragm	5	5	7	17
Brain	0	1	6	7
Foreleg	8	11	8	27
Hind leg	5	11	8	24
Liver	5	2	3	10
Other organs/tissues	5	9	8	22

^a^ *n* number of carcasses

When examining the effectiveness of dissection of different organ/tissue combinations (‘selective dissection’) instead of full carcasses for the detection of *T. solium* cysticercosis in pigs, the two equally optimal combinations were dissection of diaphragm, tongue and heart; and diaphragm, tongue and masseter. These combinations would each detect 27 of the 38 infected carcasses (71%; 95% CI: 54–85%) (Table 4). In infected carcasses with more than ten cysticerci, selectively dissecting only the diaphragm, tongue and masseters, or the combination diaphragm, heart and masseters, would have detected 19 of the 22 infected carcasses (86%; 95% CI: 65–97%) (Table 5).

**Table 4:** Number of infected carcasses identified by ‘selective dissection’ of different organ/tissue combinations for the detection of *T. solium* cysticercosis in 38 confirmed infected pig carcasses

	Heart	Tongue	Diaphragm	Masseter	Psoas
Heart	14	–	–	–	–
Tongue	21	17	–	–	–
Diaphragm	22	25	17	–	–
Masseter	19	21	21	13	–
Psoas	16	19	19	16	10
Heart and tongue	na	na	27	25	22
Heart and diaphragm	na	27	na	25	23
Heart and masseter	na	25	25	na	21
Heart and psoas	na	22	23	21	na
Tongue and diaphragm	27	na	na	27	25
Tongue and masseter	25	na	27	na	23
Tongue and psoas	22	na	25	23	na
Diaphragm and masseter	25	27	na	na	22
Diaphragm and psoas	23	25	na	22	na
Masseter and psoas	21	23	22	na	na

*Abbreviation*: *na* not applicable

**Table 5:** Number of infected carcasses identified by ‘selective dissection’ of different organ/tissue combinations for the detection of *T. solium* cysticercosis in 22 confirmed infected pig carcasses with more than ten cysts

	Heart	Tongue	Diaphragm	Masseter	Psoas
Heart	12	–	–	–	–
Tongue	15	13	–	–	–
Diaphragm	16	17	12	–	–
Masseter	16	16	16	12	–
Psoas	14	15	14	15	10
Heart and tongue	na	na	18	18	16
Heart and diaphragm	na	18	na	19	17
Heart and masseter	na	18	19	na	18
Heart and psoas	na	16	17	18	na
Tongue and diaphragm	18	na	na	19	17
Tongue and masseter	18	na	19	na	18
Tongue and psoas	16	na	17	18	na
Diaphragm and masseter	19	19	na	na	17
Diaphragm and psoas	17	17	na	17	na
Masseter and psoas	18	18	17	na	na

*Abbreviation*: *na* not applicable

### Ag-ELISA

Of the 68 dissected pigs, a total of 36/68 (53%) tested positive for *T. solium* cysticerci circulating antigens by Ag-ELISA. Of the four tongue positives, three tested positive on Ag-ELISA. Of the 38 *T. solium* dissection positive carcasses, 26 serum samples tested positive (68%; 95% CI: 51–82%). From the 30 *T. solium* dissection negative carcasses, Ag-ELISA returned ten positives, of which two were infected with *T. hydatigena* (based on dissection).

Considering the full carcass dissections as gold standard, the sensitivity and specificity of the Ag-ELISA for the detection of porcine cysticercosis (all cysticercus stages) were determined at 68% (95% CI: 51–82%) and 67% (95% CI: 47–83%), respectively. The sensitivity increased to 91% (95% CI: 71–99%) for carcasses with ten or more cysticerci. The Ag-ELISA was originally developed specifically to detect viable cysticerci as only these release antigens in the circulation [21]. If only viable cysticerci are considered, the sensitivity of the Ag-ELISA to detect carcasses with viable cysticerci (1 or more) was estimated at 91% (95% CI: 71–99%), and increased further to 100% (95% CI: 75–100%) for carcasses with at least 10 viable cysticerci (Table 6).

**Table 6:** Test performances of the Ag-ELISA according to the infection level and cyst stage in 38 confirmed infected pigs of slaughter age

*Taenia solium* infection level and stage	Ag-ELISA-positive	Ag-ELISA-negative	Sensitivity (95% CI)
Carcasses with ≥ 1 cysticercus (*n* = 38)	26	12	68 (51–83)
Carcasses with ≥ 10 cysticerci (*n* = 22)	20	2	91 (71–99)
Carcasses with ≥ 1 viable cysticercus (*n* = 22)	20	2	91 (71–99)
Carcasses with ≥ 10 viable cysticerci (*n* = 13)	13	0	100 (75–100)

**Fig. 1:**
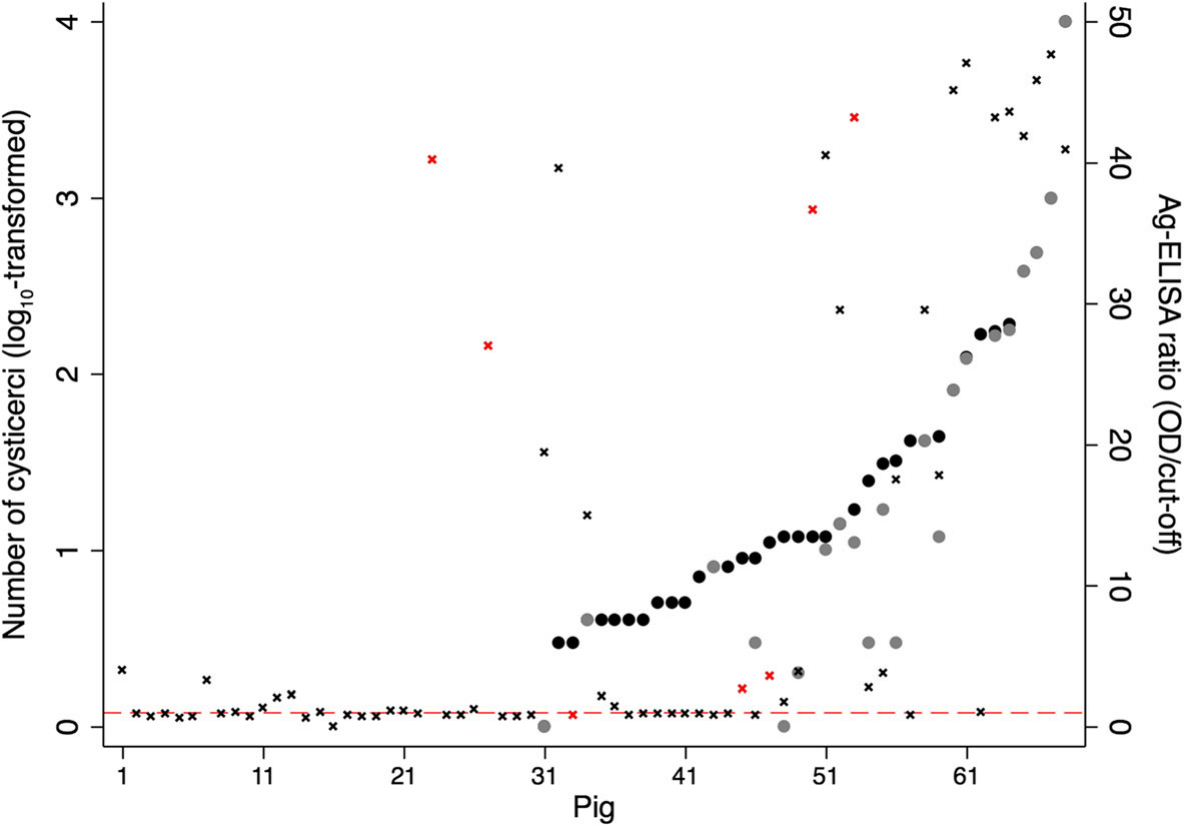
Overview of the *T. solium* cysticercosis infection level (total number of (viable) cysticerci; log_10_-transformed; total number of cysticerci indicated with black circles and number of viable cysticerci with grey circles) and Ag-ELISA ratio (OD/cut-off; indicated with x) per pig (*n* = 68). Ag-ELISA ratios in black and red indicate *T. hydatigena* negative and positive animals, respectively. The dashed horizontal line indicates the cut-off value for a positive ELISA result, i.e. ratio = 1

**Fig. 2:**
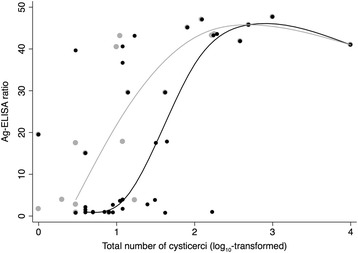
Relation between Ag-ELISA ratio and the total number of cysticerci (log_10_-transformed; *n* = 38 *T. solium* positive animals; indicated in black) and the total number of viable cysticerci (*n* = 22 confirmed *T. solium* infected carcasses with viable cysticerci; indicated in grey). The curves represent the median spline plots, which use the cross medians to fit a cubic spline. Stata/IC 14.1 (StataCorp, College Station, TX, USA)

Figures 1 and 2 present the Ag-ELISA ratios (OD/cut-off) in relation to the infection levels detected in the carcasses. In *T. solium* dissection positive carcasses, the Ag-ELISA ratios were higher in pigs with ≥ 100 cysticerci (*n* = 8) than in pigs with 11–99 cysticerci (*n* = 14; *Z* = 2.525, *P* = 0.012), and the latter group had higher ratios compared to pigs that had ≤ 10 cysticerci (*n* = 16; *Z* = 3.016, *P* = 0.003). Similarly, pigs with ≥100 viable cysticerci (*n* = 7) had higher ratios than pigs with 11–99 viable cysticerci (*n* = 6; *Z* = 2.143, *P* = 0.032), which had higher Ag-ELISA ratios than pigs with ≤ 10 viable cysticerci (*n* = 9; *Z* = 2.121, *P* = 0.034). An overall significant increasing trend was found across the groups for both total and viable cysticerci (*Z* = 4.30 and *Z* = 3.72, *P* < 0.001). Ratios from the ten non-infected carcasses that tested positive by Ag-ELISA were generally low (1.10–3.98), except for two carcasses in which *T. hydatigena* cyst(s) were detected (ratio 27.02 and 40.19). *T. hydatigena* cyst(s) were detected in seven of the 68 dissected carcasses, of which six tested positive on Ag-ELISA (ratios ranging between 2.64–43.13) and one tested negative (ratio 0.79).

### Molecular identification of cysticerci (PCR-RFLP)

For all cysticerci morphologically identified as *T. solium*, one cysticerc collected from the muscles of each carcass (38) and at least one cysticerc collected from each organ per carcass (liver: 10; spleen: 1; lungs: 2) was analysed. All the analysed cysticerci from the 38 *T. solium* dissection-positive carcasses (muscle tissues and other organs, including the very small cysts) were confirmed by PCR-RFLP as *T. solium*. All *T. hydatigena* cysts identified morphologically at dissection were confirmed by PCR-RFLP as *T. hydatigena.*

## Discussion

This study recorded a high prevalence (56%) of porcine cysticercosis in the pigs studied in the Eastern Province of Zambia, based on half and full carcass dissections of randomly selected pigs of slaughter age. This figure is in the same magnitude as the earlier 64% prevalence estimation of porcine cysticercosis in Zambia based on a Bayesian analysis of serological, tongue palpation and routine meat inspection results [6, 11].

The fact that 56% of the slaughter-aged pigs studied were infected with this zoonotic parasite despite the intervention methods employed by various stakeholders (e.g. measures to improve sanitation) is worrying for public health. This is of additional concern given that more than half of the infected carcasses (58%) had viable cysticerci. The cysticerci present in the meat of the randomly selected pigs of slaughter age in this study would have potentially entered the food chain, with 87% of the cysticerci viable (infectious). In studies in Cameroon and Peru, 19.6 and 16.8% of the carcasses were infected based on (half) carcass dissection, respectively. Infection levels differ between these countries with 20% of the infected pigs in Cameroon having less than 50 cysticerci, while this was 50% in Peru [22]. In our study, most carcasses (76%) had 1–50 cysticerci.

Tongue palpation detected 4 of the 38 (11%) dissection positives, confirming its low sensitivity for detecting porcine cysticercosis [6]. Indeed, in a previous study conducted in Zambia, tongue palpation detected 7.7% of infected pigs, while meat inspection detected 18.5% of infected pigs (infection status determined by carcass dissections) [11]. Half carcass dissection, often used as a substitute for full carcass dissection, would have detected 84% of the infected carcasses in our study, representing a certain loss in sensitivity (Se), while the workload still remains substantial. An alternative described by Lightowlers et al. [22] suggested dissecting only the tongue, heart and masticatory muscles, which, in their study including animals from Cameroon and Peru, led to a detection of 81% of the infected animals. In our study, dissection of these organs would have led to the detection of 66% of infected carcasses only, suggesting that the effectiveness of this approach might vary, and is likely dependent on infection intensity. Also, in the study of Lightowlers et al. [22], carcasses from animals from Cameroon were only half dissected, which, according to our results can lead to a non-detection of 16% of cases, and as such affect the ‘gold standard’. Including the diaphragm, tongue and masseter led to a detection of 71% of cases in our study, increasing to 86% in carcasses infected with ten or more cysticerci. Of course, choice of organs/muscle groups that can be dissected will be influenced by other aspects such as size and price of the organ, ease of slicing and recognising the cysticerci, consumers’ preferences amongst others. Whether a sensitivity of 71% for this ‘selective dissection’ is sufficient and a better option than the use of serological tools is debatable, and largely dependent on the aim of detection.

A similar sensitivity was observed for the Ag-ELISA, a much more user friendly and cheaper technique, which can be applied ante mortem and will as such not impact ongoing interventions. The main drawback is its low specificity (67%), though this can be resolved by (selective) dissections of the positives. The latter should be completed by a thorough investigation for presence of *T. hydatigena* cysts. When the aim is to reduce the risk for public health, that is removal of infectious (viable) cysticerci from the food chain, the use of the Ag-ELISA would be more effective with sensitivities of 91% for carcasses with 1 or more viable cysticerci, and 100% for carcasses with more than 10 cysticerci. Lack of sensitivity and specificity has been described for antibody detecting serological tools as well [13]. The routinely used EITB for detection of human (neuro)cysticercosis, fails to deliver the same performance in pigs, where sensitivities and specificities have been re-assessed at 89% and 43%; respectively [12]. In a recent initiative, the Target Product Profile (TPP) for the detection of porcine cysticercosis had been initiated at a WHO stakeholder meeting, and was further finalised after consultation with 53 stakeholders [23]. The TPP indicates the need for a test that at least detects specifically *T. solium* porcine cysticercosis, only viable cysts and preferable in blood spots collected on filter paper. The minimal clinical sensitivity demands were set at 50% for less than 50 viable cysts and 80% for more than 50 viable cysts. The minimal clinical specificity was set at 95%. An additional remark was the need for validation in endemic areas to assess cross-reactions. Indeed, besides the known fact that the Ag-ELISA used in this study cross-reacts with *T. hydatigena*, false positive results due to exposure to other *Taenia* spp. were hypothesized [24]. However, in a recent study where piglets were orally infected with *Taenia saginata* eggs, no specific antibodies (as measured by a commercial western blot) or specific circulating antigens (measured with the B158/B60 Ag-ELISA) could be detected [25], challenging that hypothesis.

An important finding from our study is the presence of *T. solium* cysticerci in the liver, spleen and lungs, locations that are not routinely included in full carcass dissections. Also, very small (viable) *T. solium* cysticerci were observed in organs and muscles, which could easily be missed, even when slices of 3 mm are performed. These findings question the true value of full carcass dissection (with the recommended 5 mm slices) as a gold standard used for the evaluation of interventions and diagnostic tools. Indeed, in our study, this factor may have led to an underestimation of the true specificity of the Ag-ELISA.

The observation of mainly viable cysticerci in the livers of 26% of the infected carcasses may have important consequences, as in certain cultures, the liver is eaten raw or insufficiently cooked. This finding should be considered in evaluations of control programmes, and also in public health messaging.

## Conclusions

In conclusion, our findings indicate a very high occurrence of porcine cysticercosis in the study area. Full carcass dissection is still recognized as the most sensitive diagnostic tool for the detection of porcine cysticercosis, however dissection of the liver, spleen, kidneys and lungs should be included, and results should be interpreted carefully as small cysticerci can easily be overlooked, leading to false negative results. As an alternative, ‘selective dissections’ of tongue, diaphragm and masseter or heart muscles could be envisaged, although this does not necessarily lead to better sensitivities than circulating antigen detection. The latter’s main drawback is its lack of specificity. The most optimal and most efficient approach will depend on the aims of the diagnosis and might include a combination of both serology and selective dissection of the serology-positives. Other alternatives, such as ultrasound [26], should be further investigated.

## Abbreviations

Ag-ELISA: Antigen detecting enzyme linked immunosorbent assay
CI: Confidence interval
EITB: Enzyme-linked immunoelectrotransfer blot
HH: Household
Na: Not applicable
OD: Optical density
OR: Odds ratio
PCR-RFLP: PCR-restriction fragment length polymorphism
Se: Sensitivity
Sp: Specificity
